# Economy of Labour in Hospital Management

**Published:** 1912-07-13

**Authors:** Alfred M. Hooper


### ECONOMY OF LABOUR IN HOSPITAL MANAGEMENT.

By ALFRED M. HOOPER.

The administration of every charitable institution should be based on efficiency and economy. The latter principle applies not only to the direct expenditure of money in purchase of provisions, medical and surgical requirements, and other numerous necessities, but also to the indirect, but nevertheless heavy, outlay in the shape of labour.

Much has been written with regard to the direct expenditure, and useful comparison has become possible by means of the "Uniform System of Hospital Accounts," introduced by "King Edward's Hospital Fund" and "The Saturday and Sunday Funds," now generally adopted. This has, doubtless, been the means of effecting reduction in expenditure.

Economy in this direction, unfortunately, is under our control to only a limited extent. The market value of provisions, as is well known, has risen enormously during the past few years, and it is probable that the unrest in labour circles will tend to still more enhanced prices. New instruments and appliances are constantly being brought into use, and every hospital must keep its equipment in the best possible state of efficiency.

When, however, we consider the expenditure on labour we find that it is more or less subject to the economic methods we employ. Every hospital has its own special methods of administration and system for the execution of the routine work of its office, but the principle underlying their methods is practically the same in every institution, differing only in detail.

Of these methods, none is more practical, from the point of view of economy, than the practice of using printed forms—whether it be with the object of giving instructions to patients, nurses, domestic servants, orders to employees, information to enquirers, or any of the many other purposes for which it is applicable.

The value of the "printed form" depends largely on its suitability for the purpose intended. It should be as short as possible, and yet easily comprehensible.

In every hospital there must be a large number and variety of "Forms" in use, and probably there exists a great diversity in the character of those used for the same purpose in different institutions. I propose to give particulars of some of those in use at one London hospital, and evolved without reference to those existing at other institutions. First, I will deal with the notification of deaths. It is the custom for the secretary, or his representative, to interview the relatives of all deceased patients, to explain to them the regulations and necessary steps to be taken to register the death, and to endeavour to obtain sanction for a post-mortem examination. The record of such interview is entered on a card, which is filed; so that, in the event of any question arising, evidence is available as to the interview with the relatives and the result, the card being initialed by the officer responsible. Immediately on the death of a patient, it is the duty of the sister in charge of the ward to fill in the upper part of the card with the name of the ward, the patient, and the time of death, and deliver it to the secretary's office. Upon permission for an examination being obtained, the papers connected with the case are handed to the pathological department, and no post-mortem may be made until this has been done. The border of the card is black; this not only serves as complimentary mourning but makes it the more readily distinguished from the many other cards in use.

The second form used in the same connection is a notice to the relatives of the deceased, giving instructions as to registering the death and an authority to the undertaker to remove the corpse. The mental condition of relatives, following their loss, is often such as to render it a somewhat difficult matter to make them thoroughly understand the necessary steps to be taken, and this form is found to save a good deal of verbal explanation. The "Undertaker's Authority" is perforated for removal, and is found to be of great service in doing away with the possibility of unauthorised removal of the body, and it also serves as a receipt.

The "Office Instruction Book" is used to issue orders to the mechanic, carpenter, and other employees. The book is made with duplicate sheets used with a carbon paper; the original is perforated, and when filled in, detached and handed to the employee, whose duty it is to return it immediately the work is completed. We thus have a record of every order issued, can ascertain what work is outstanding, the time any particular piece of work has taken to complete, and a useful record of how the men's time is employed.

The "Goods Order Book" is made with duplicate sheets and used with carbon, and can hardly require any explanation. The printed condition that the order must accompany the quarterly statement of accounts is not strictly adhered to, as it would be obviously unfair to make the supplier of goods part with his authority; it is, however, useful in the case of any question as to the actual terms of the order, especially so when there are several officers authorised to order goods.

The cards from the "Contribution Filing Cabinet" contain the name and address of the contributor, particulars as to the method of acknowledgment, etc., the due date, the date paid, and the amount. The index used is a very full one, and enables us to look up at once any contributor, no matter under what *nom de plume* he chooses his contribution to be entered. Having the cards arranged alphabetically is of great assistance in compiling the list of contributions for the annual report.

The "Recommendation Form" for the provision of surgical appliances out of the Samaritan Fund is signed by the surgeon. It requires little explanation, except that, although the surgeon may recommend the aid of the Samaritan Fund, the ultimate decision as to whether the patient is eligible for such assistance rests with the office authorities.

It may be mentioned that numbers are printed in the left-hand bottom corner of the "Forms" denoting, first, the number on the schedule of prices, then the number of the last order and month and year of last supplies.

These are a few examples of the various "Forms" we have found very serviceable in the administration department of our hospital, and I hope others may be induced to give their experience in dealing with similar matters, with which every hospital secretary has to cope. I think that if some recognised set of "Forms" were drawn up and universally adopted by hospitals it would tend to the perfection in the system of management, economy of labour, and reduction in expenditure.

[We shall be pleased to forward a copy of each of the forms mentioned in the article to anyone interested who will send us a stamped addressed envelope.—Ed. "The Hospital."]

